# Health systems research for policy change: lessons from the implementation of rapid assessment protocols for diabetes in low- and middle-income settings

**DOI:** 10.1186/s12961-015-0029-4

**Published:** 2015-10-01

**Authors:** David Beran, J. Jaime Miranda, Maria Kathia Cardenas, Maryam Bigdeli

**Affiliations:** Division of Tropical and Humanitarian Medicine, Geneva University Hospitals and University of Geneva, Geneva, Switzerland; CRONICAS Center of Excellence in Chronic Diseases, Universidad Peruana Cayetano Heredia, Lima, Peru; Alliance for Health Systems and Policy Research, World Health Organization, Geneva, Switzerland

**Keywords:** Diabetes, Health systems, Health systems research, Policy

## Abstract

**Background:**

As many challenges exist for access to diabetes care in developing countries, the International Insulin Foundation developed a Rapid Assessment tool and implemented this approach to identify barriers to care and propose concrete recommendations for decision makers. The objective of this paper is to identify the factors that contributed to informing and influencing policymakers with regards to this work.

**Methods:**

A documentary review comprised Stage 1. Stage 2 used an online questionnaire to gain insight from users of the Rapid Assessment results. Based on Stages 1 and 2, Stage 3 comprised in-depth interviews with a total of nine individuals (one individual each from the six participating countries; two individuals from the World Health Organization; one “Global Diabetes Advocate”). Interviews were analyzed based on a list of themes developed from Stage 2.

**Results:**

Stage 1 led to the identification of various types of documents referring to the results. The online questionnaire had a response rate of 33%. Respondents directly involved in the assessment had a “Good” or “Very Good” appreciation of most aspects and scored these higher than those not directly involved. From the interviews, formalized methods and close collaboration between the international team and local partners were strengths. Trust and a relationship with local partners were also seen as assets. All stakeholders valued the results and the credibility of the data generated. Local partners felt that more could have been done for dissemination.

**Conclusion:**

This study shows the importance of specific results from the different assessments. In addressing complex issues having external experts involved was seen as an advantage. The uptake of results was due to the credibility of the research which was influenced by a mix of the people involved, past assessments, trusted local partners, and the use of the results by knowledge brokers, such as the World Health Organization. Through these brokers, others gained ownership of the data. The methods used and the fact that this data was grounded in a local context also reinforced its value. Despite limitations, this study offers a unique perspective where a similar research approach was taken in six countries.

## Background

Diabetes, which led to 1.3 million deaths in 2010 [1], is one of the four conditions prioritized by the World Health Organization (WHO) in its Global Action Plan for the prevention and control of non-communicable diseases (NCD) 2013–2020 [2]. Access to diabetes care in developing countries is described as being problematic with most of the factors documented relating to access to medicines and especially insulin [3-5]. Other issues related to the health system, healthcare worker training, and access to diagnostic facilities and syringes, are known to also have a negative impact in availability and provision of diabetes care, but are not widely described.

To address this problem, the International Insulin Foundation (IIF) developed the Rapid Assessment Protocol for Insulin Access (RAPIA) [6]. The RAPIA is structured as a multi-level assessment tool for health systems research. It studies the different elements that influence access that people with diabetes have to care in a given country. The data collection process provides a country-specific situation analysis regarding diabetes care including access to medicines. This approach also highlights the strengths and weaknesses of the health system in proposing concrete actions [7-9]. To date, the RAPIA has been implemented by the IIF in six countries, spanning four WHO Regions: Kyrgyzstan, Mali, Mozambique, Zambia, Nicaragua, and Vietnam [10-15]. Table 1 describes the main elements of the RAPIA.

**Table 1:** **Key components of the RAPIA [** 6 **]**

-	Data collection tools tailored to different levels of the health system and key informants
	○ Purposive and convenience sampling
	- Multiple methods of data collection
	- Multiple data sources
	○ Interviews
	▪ Ministry of Health, health professionals, health facilities, traditional healers, individuals with diabetes, etc.
	○ Site visits
	▪ Three areas of the country
	• Capital City
	• Urban area
	• Predominantly rural area
	○ Document reviews
	○ Use of existing statistics
-	Cyclical process – data collected informs further data collection
-	System perspective
-	Triangulation of:
	○ Data sources
	○ Perceptions
	○ Research methods

In these six countries, the RAPIAs were the first NCD- or diabetes-specific research carried out. Besides highlighting the barriers to care, the RAPIA helped estimate life expectancy, the burden of disease, and the cost to the health system. From a policy perspective, the RAPIA resulted with diabetes being included as part of the existing cardiovascular disease program in Kyrgyzstan, thereby expanding the area of NCDs within the country’s national health program [16]. In Mozambique, the RAPIA recommendations were incorporated into an overall NCD Strategy [17] rather than being diabetes specific.

Gilson [18] defines health policies as “*actions through which efforts are made to strengthen health systems in order to promote population health*”, but as stated by the WHO, very little is known about how to facilitate the use of research in developing countries [19]. Hanney et al. [20] state that a gap in the research in the area of using health systems research in policy is linked to the understanding of how research is used, and as discussed by Hyder et al. [21], this is linked to the interface between research and policy. Therefore, the objective of this paper is to analyse the RAPIA implementation and the factors that contributed to informing and influencing policymakers in Kyrgyzstan, Mali, Mozambique, Nicaragua, Vietnam, and Zambia.

## Methods

The first stage included a documentary review to identify peer-reviewed publications, reports, and other published materials, which included the results of the RAPIA. For peer-reviewed publications Web of Knowledge and PubMed were used to assess the number of citations these articles had. In addition, for all open access articles the number of accesses was found.

As the objectives of the IIF’s research was not only to inform academic audiences, but were rather targeted at policymakers, a general search strategy using Google was developed. This search strategy used the search terms shown in Table 2, restricted to the years 2003 until 1 May 2013. Each term was combined using the term connector “AND”, for example “International Insulin Foundation” AND “NCD Alliance”. In addition, the title of the RAPIA report in French (Mali), Portuguese (Mozambique), Spanish (Nicaragua), Russian (Kyrgyzstan), and Vietnamese (Vietnam) was entered into a Google search engine. The aim of this search was to identify documents that had used or referenced material produced by the IIF using the RAPIA tool.

**Table 2:** **Search terms for Google search**

**Search terms**	**NCD Alliance**	**IDF**	**WHO**	**UN HLM**	**Mozambique**	**Zambia**	**Mali**	**Nicaragua**	**Vietnam**	**Kyrgyzstan**
International Insulin Foundation										
Rapid Assessment Protocol for Insulin Access										
Yudkin										
Beran										

IDF - International Diabetes Federation.

WHO - World Health Organization.

UN HLM - United Nations High Level Meeting on Noncommunicable diseases.

Stage 2 used the results from the above search to develop a list of individuals who had used or referred to results from the different RAPIAs. These included authors of peer reviewed publications, reports, and news items. In addition, the IIF kept a list of individuals who had contacted it about the RAPIA in reference to the tool or its results and these people were also added to this list. All these people were sent an introductory e-mail describing the study and a link to a web-based questionnaire, which included a 3-week response period. After 2 weeks, a reminder was sent. The aim of the questionnaire was to assess the perceptions of these individuals with regards to which elements of the RAPIA helped impact on policy. This survey was conducted between March 26 and April 12, 2013. The questionnaire had a total of 27 questions with a mix of Likert scales (Very Poor to Very Good and Strongly Disagree to Strongly Agree) giving scores from 0–5 and areas where individuals could add their comments. The first questions tried to gain some background information on the respondent. People then had to answer if they had knowledge of the IIF or not. Those with knowledge of the IIF were asked if they were directly involved in the implementation of a specific RAPIA. Those who were not directly involved in the RAPIA were asked how they came to know about the IIF’s work. Those who stated that they were unaware of the IIF’s work were asked if they knew about the work of individuals involved with the IIF. The questionnaire then asked questions looking at aspects of quality related to (1) methods used in the RAPIA; (2) quality of the research team; (3) credibility of the research team; (4) involvement of local partners during the research process; (5) quality of the results from the RAPIA; (6) quality of the recommendations from the RAPIA; (7) quality of the dissemination of the RAPIA results; and (8) priority setting exercises. The answers from these questions helped develop the items for discussion used in the next stage.

A more in-depth look at what some key stakeholders thought of the RAPIA and IIF’s work was carried out during Stage 3 using the information collected from Stages 1 and 2. One of the main partners in each of the six countries where the RAPIA was carried out, except in Zambia where two were included, was contacted and asked if they were willing to participate in an interview. In addition, two WHO officers, one from Headquarters and the other from a country office, and a Global Diabetes Advocate active in the area of access to insulin were also interviewed. These individuals were selected due to their close links with the implementation of the RAPIA in each country or knowledge of the work done by the IIF from a more global perspective. Interviews were carried out by DB on the phone or Skype, in May 2013, and lasted on average of 46 minutes. Detailed notes were taken during each interview. Thematic analysis was carried out using the headings from a discussion guide. Using answers from the online questionnaire a discussion guide was developed focusing on:

- RAPIA Methods
- Research team and collaboration
- Results from the RAPIA implementations
- Dissemination of RAPIA results
- Recommendations from RAPIA reports

This research proposal was submitted to the Ethics Board at the Geneva University Hospitals.

## Results

### Documentary review

Table 3 presents the citations and accesses (where publications enabled authors to see how many times their articles have been viewed online) of the different peer reviewed publications published as a result of the RAPIA. The results from the Google search found that organizations such as the Hanoi School of Public Health, Health Action International, International Diabetes Federation, NCD Alliance [22], NCD Child [23], Novo Nordisk [24,25] (leading insulin manufacturer), RAND Health, the World Bank [26], and WHO at Headquarter, Regional, and National levels (summarized in Table 4), either referenced the publications detailed in Table 3 or one of the RAPIA country reports. The WHO Essential Medicines and Health Products website also includes a list of publications comprising the results from the different RAPIA implementations [27].

**Table 3:** **Peer reviewed publications on the results of the Rapid Assessment Protocol for Insulin Access or the International Insulin Foundation work**

**Title**	**Journal**	**Citations (Web of Knowledge)**	**Google scholar**	**Accesses**
Non-communicable diseases in Mozambique: risk factors, burden, response and outcomes to date	Globalization and Health	0	0	1,867
Improving access to insulin: what can be done?	Diabetes Management	NR	2	NR
The insulin dilemma in resource-limited countries. A way forward?	Diabetologia	8	10	NR
The Diabetes UK Mozambique Twinning Programme. Results of improvements in diabetes care in Mozambique: a reassessment 6 years later using the Rapid Assessment Protocol for Insulin Access	Diabetic Medicine	4	6	NR
Looking beyond the issue of access to insulin: What is needed for proper diabetes care in resource poor settings	Diabetes Research in Clinical Practice	5	10	NR
Twinning for better diabetes care: a model for improving healthcare for non-communicable diseases in resource-poor countries	Postgraduate Medical Journal	4	7	NR
Access to medicines versus access to treatment: the case of type 1 diabetes	Bulletin of the World Health Organization	5	14	NR
Le diabète: un nouvel enjeu de santé publique pour les pays en voie de développement: l’exemple du Mali.	Médecine des maladies Métaboliques	NR	10	NR
Diabetes Care in sub-Saharan Africa	Lancet	44	99	NR
Assessing health systems for type 1 diabetes in sub-Saharan Africa: developing a ‘Rapid Assessment Protocol for Insulin Access’	BMC Health Services Research	11	21	5,297
Access to care for patients with insulin-requiring diabetes in developing countries: case studies of Mozambique and Zambia.	Diabetes Care	24	60	NR
Prognosis of diabetes in the developing world	Lancet	2	4	NR

NR, Not referenced.

**Table 4:** **Use of International Insulin Foundation materials by the World Health Organization**

**Level of WHO**	**Title of document**
Headquarters	Prioritized Research Agenda for Prevention and Control of Noncommunicable Diseases [44]
Headquarters	Essential Medicines for Noncommunicable Diseases [45]
Headquarters	Equity, social determinants and public health programmes [46]
Headquarters	Noncommunicable Diseases, Poverty and the Development Agenda [47]
WHO Regional Office for Africa	WHO African Region Ministerial Consultation on Noncommunicable Diseases background document [48]
WHO South-East Asia Regional Office	Technical report on Social disparities in health in the Maldives [49]
WHO Country Office Kyrgyzstan	Report included on website [50]
WHO Country Office Vietnam	Medicines Prices: Policy options for Vietnam [51]

The Google search also identified news items from different mass media around the world, such as the BBC [28], Guardian [29], Financial Times [30], Asia Life [31], and the British Medical Journal [32], which referenced the RAPIA results or included interviews with people involved with the IIF discussing findings from the RAPIAs and their wider implications. During this overall Google search it was found that 15 textbooks also referenced IIF publications.

### Online questionnaire

**Figure 1:**
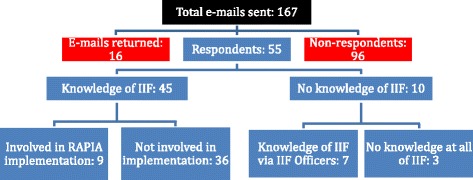
**Details of responses to online questionnaire**

The questionnaire was sent to a total of 167 people, with individuals in each country where the RAPIA had been implemented representing 66 possible respondents and 85 individuals seen as international partners; international partners may have collaborated with the IIF or cited its work, but were not directly involved in a specific assessment. Figure 1 details the response to the overall questionnaire. The overall response rate was 33% (n = 55). Only nine out of the 55 respondents stated they were directly involved in the implementation of the RAPIA. Of these, two were from Kyrgyzstan, one from Mali, three from Mozambique, zero from Nicaragua, two from Vietnam, and one from Zambia. Responders who stated that they were not directly involved in the implementation of an in-country assessment (n = 36), had heard about the IIF through peer reviewed publications (53%), seen a presentation about this work (53%), read about this work but not in a peer reviewed publication (33%), or in an different way (19%), including mainly meetings and being informed about this work through colleagues as well as the IIF’s website. For those who stated they were unaware of the work of the IIF, they were asked if they knew about the work of the Trustees and Advisor to the Board of the IIF regarding access to insulin. Overall, 80% of these individuals responded positively and 86% had heard about this work from a presentation given by either of these individuals.

The answers show that those who stated that they were directly involved in the RAPIA in each country had a “Good” (score of 4) or “Very Good” (score of 5) appreciation of most aspects of the RAPIA. Overall, those stating that they were directly involved scored each of these elements higher than those stating they were not directly involved. For those stating that they were not involved, the quality of the research team and results was scored lower than by those stating that they were directly involved. Both groups viewed dissemination as the weakest element (Table 5). In looking at some of the comments left with regards to dissemination, one respondent stated, “*The work of the IIF has an evidence-informed body of work that could be better leveraged to influence policy*”. Another respondent highlighted this issue for dissemination of results at an international level saying that the “*IIF has been preaching to the converts, i.e. the diabetes community*”.

**Table 5:** **Rating of different elements of Rapid Assessment Protocol for Insulin Access (RAPIA) process by respondents directly and not directly involved in local assessments**

**Element of the RAPIA**	**Directly involved**	**Not directly involved**
Methods used in the RAPIA	4.9	4.8
Quality of the research team	4.9	4.6
Credibility of the research team	4.9	4.7
Involvement of local partners during the research process	4.8	4.7
Quality of the results from the RAPIA	4.9	4.6
Quality of the recommendations from the RAPIA	4.8	4.7
Quality of the dissemination of the RAPIA	4.4	4.2

### Interviews

From the 10 people contacted, nine were interviewed. The analysis of the themes yielded the following insight.

#### RAPIA methods

The Global Diabetes Advocate and Vietnamese partner stated that a strength of the RAPIA was that it provided “*a formalized approach*” to documenting the situation with regards to diabetes care and access to insulin in low- and middle-income countries. The Vietnamese partner added that other strengths were that the RAPIA used methods, which were “*pre-packaged*” and had already been used elsewhere. Partners from Kyrgyzstan, Nicaragua, and Zambia, as well as the WHO Country Officer, highlighted the RAPIA’s comprehensive structure studying all levels of the health system. The Kyrgyz and Nicaraguan partners added that another of the RAPIA’s strengths was its ability to adapt to the specific context and health system in different countries.

The WHO Country Officer, in describing the methods and their use, stated that the “*methods are quite good as it not only provides availability and affordability information, but also health system barriers to diabetes care*”. This person went on to add that “*prices and availability surveys do not include context – RAPIA does as it provides information about: the different levels of the health system; human resources; financial aspects and overall context. It gives an overview of diabetes treatment as a whole and not only medicines*”.

An interesting point raised by the Kyrgyz partner was that, as the methods require site visits and interviews at different levels of the health system, this created “*noise about the study*” and therefore got people interested in the study early on.

#### Research team and collaboration

The partners in Kyrgyzstan, Mali, Mozambique, and Vietnam mentioned the close collaboration between the IIF and local partners as a strength of the research team. The WHO Country Officer stated that the IIF was able to build trust and a relationship with local partners and address difficult issues around access to medicines and care. The partner in Vietnam stated that “*looking at access to medicines and insulin is complex*” and that capacity to do this in low- and middle-income countries is limited and that this is what the IIF brought. The contribution by the local partner was an understanding of the local situation and the right people to help. In Mali, the partner also added that having an external person (DB) allowed for the RAPIA to be “*critical*”, which may not have been possible for an internal team on its own.

#### Results from the RAPIA implementations

All individuals interviewed highlighted that the RAPIA was the first study of its kind to be carried out in these different countries and all stakeholders involved valued it. The WHO Country Officer stated that the issue of affordability and access to medicines is controversial, but important, and the scientific approach and involvement of local partners in the data collection helped with the ownership of the data and the use of this data locally. In Vietnam, the partner stated that the results and report were read by many and this was useful to different people and not only policymakers as the wealth of results was useful for everyone.

Interviewees from Kyrgyzstan, Mozambique, and Vietnam stated that the RAPIA results had an impact on policy. In Mozambique “*data collected by the RAPIA showed that diabetes was a public health problem in Mozambique and was used in the best possible way as this was included in the NCD Strategic Plan, National Health Policy, Government Plans and project proposals*”. The results from the RAPIA were included in a strategy on diabetes in Kyrgyzstan and were seen as “*very important for policy development*” in Vietnam.

The WHO Country Officer, in describing why the RAPIA results had an impact on policy development, stated that this was due to the specific data collected on the affordability of medicines, the high financial burden on individuals, and especially that “*prices for Essential Medicines were high and unaffordable*”. This aspect was also highlighted by the partner in Nicaragua in terms of medicines, but also globally for the issue of diabetes and provided “*evidence of what was there and what was happening*”. The partner in Zambia also stated that it “*showed people involved* [in diabetes] *the challenges*” adding that this was specifically with regards to insulin access and healthcare worker training.

One piece of information highlighted as having a large impact by the partners in Mozambique and Vietnam, as well as the Global Diabetes Advocate, was the calculations the IIF made using the RAPIA on life expectancy for people with Type 1 diabetes. The Global Diabetes Advocate mentioned that this data was useful despite its serious assumptions. They also highlighted that the RAPIA was able to quantify issues that people knew about, but did not have data on, for example, travel costs.

Another useful aspect of the results from the RAPIA that was discussed by the WHO Country Officer was that statistics on affordability exist, but that the RAPIA gave family stories and a personal perspective to the issue. They stated that these small factual stories made the difference and “*for policymakers 5–10% are interested in the science, but practical examples have much more impact*”.

In terms of credibility, there was the credibility of the data generated by the IIF through the RAPIAs, but also the credibility of the IIF as an organization. From the perspective of the Global Diabetes Advocate, some of the IIF’s positions on access to insulin were detrimental. They stated that there was the need to sometimes set aside “*personal views versus larger picture*” and the need to avoid being dogmatic. However, these same positions were what gave the partner in Vietnam the confidence and interest in working with the IIF as they viewed the IIF as an organization with “*integrity and objectivity*”.

#### Dissemination of RAPIA results

In looking at the dissemination, the partners from Nicaragua and Mozambique felt that more “*noise*” could have been made during the dissemination and a wider spectrum of people included in the formal dissemination of the report. The partner in Mali echoed this and said the dissemination went very well, but more could always be done. The partner in Vietnam added that it was also felt that more people should have been included in the dissemination as well as more time with beneficiaries, i.e. people with diabetes to explain the results to them.

The Kyrgyz partner was extremely positive about the dissemination in terms of the roundtable and the discussion with policymakers. They mentioned that the initial reaction was not a positive one as local stakeholders felt that the results were negative, but after the dissemination and discussion they really appreciated the report and found the results very important. Another positive comment from Kyrgyzstan was the holding of a closed door meeting with the Ministry of Health before the wider dissemination to address some issues and present initial findings; it was felt that this added importance to the results. Another factor that added importance to the results was the presence of international experts at the workshop where the RAPIA results were presented.

In the country where the WHO Country representative worked, the dissemination continued beyond the formal presentation of the RAPIA results with the WHO and other local partners taking ownership of the results and using them in policy briefs and a presentation to the national assembly in 2010. This person highlighted that the formal dissemination helps in different stakeholders coming together, but that “*one-off dissemination is not enough and this needs to be sustained*”. In Mozambique, this was seen as possible as the local partner was viewed as a “*champion*” for NCDs and was able to use the data to move their agenda forward; for example, some of the data collected on poor distribution of insulin to regional medical stores and low life expectancy of children with diabetes. From the perspective of the WHO Officer at Headquarters “*together with Health Action International the data from the IIF’s work is widely quoted and used in looking at the issue of access to medicines for NCDs*”. The interviewees mentioned the IIF’s reports and publications in terms of dissemination of the results from the different RAPIAs.

#### Recommendations from RAPIA reports

All partners stated that the recommendations proposed by the RAPIA were extremely useful to in-country partners. In Mozambique, “*the recommendations provided in the report of the assessment were simple and clear for the Ministry of Health and they felt that they could implement these recommendations*”. In Nicaragua, “*authorities responded positively to the recommendations*”. The partner in Vietnam stated that the recommendations were “*clear, useful, helpful, offer new light*” on a variety of inter-related issues regarding access to diabetes care. In Kyrgyzstan, the recommendations served as a basis for a strategy and program on diabetes. The WHO Officer at Headquarters asserted that “*recommendations adapted to the local context were a strength of this process and allowed countries to make small, but significant changes as to how they approached the issue of diabetes*”.

From a global perspective, the Global Diabetes Advocate stated that the recommendations were “*straight forward and useful on a global level*”. They added, though, that the approach and views of the IIF sometimes hampered the implementation of these.

### Limitations

Methods for policy analysis and impact are increasingly diverse [33]. Some use qualitative methods [34], document reviews, and key informant interviews using an interview guide [35], and others suggest bibliometric analyses and how this data is included in other studies, through documentary analysis and interviews [20]. The approach taken here was to combine methods several methods [19,20,34,35]. This research is really a snapshot and it was often hard to disassociate what was impacted by the RAPIA and how this was done. For the document review, any limits in the search strategy and also documents published but unavailable freely on the Internet, may mean that key impacts of the RAPIA were missed.

Overall, the low number of local respondents and low rate of response from local partners to the online questionnaire, the main people involved in the RAPIA process, limits the assessment of what truly happened on the ground. Further, since many of the individuals included had referenced the RAPIA or contacted the IIF about its work, this clearly represents a source of bias. In reviewing response rates for online questionnaires from different studies, Dobrow et al. [36] found that they ranged from a minimum of 27.3% to maximum of 39.8% with a best estimate of 32.8%. Nulty [37], in a similar review, found that online surveys achieved response rates that were much lower than paper-based ones (on average, 33% compared with 56%). Therefore, the response rate to this survey can be seen as relatively high.

The selection of interviewees for the in-depth interviews was performed using a convenience sample and as the respondents knew the main researcher and had worked with him this may have led to some bias. As stated by Woelk et al. [35], in looking at translating research into policy, this type of research is influenced by the respondents, their role at the time of the study, and their relationship to the researcher. Many of the partners were self-selecting at the time of the study due to their interest in the topic, this already establishes a pre-existing bias. In addition, as some of the collaborations were 10 years ago, the issue of recall bias may also play a role. Further, in some countries there may have been ongoing collaborations whereas, in others, the RAPIA was the only joint project, which may have also influenced responses.

## Conclusion

Overall, as described in this project by the interviewees and by Panisset et al. [38], the RAPIA helped to map the context, identify barriers and their determinants, and propose practical solutions and recommendations. Specific results from the RAPIAs were viewed as important, such as the calculations of life expectancies and also highlighting the issue of access to diabetes care and insulin from a personal view. Getting the perspective from the Officer from the WHO Headquarters helps in assessing the impact of the RAPIA and IIF’s work. This person stated that “*The material produced from the different implementations of the RAPIA have been included in various WHO documents and helped develop Regional and Global Action plans on the issue of access to medicines for NCDs*”. This was possible according to this individual as the research and advocacy from the IIF informed WHO policy with regards to the issue of access to insulin and this material was also included in documents produced by the International Diabetes Federation and NCD Alliance in the run-up to the UN High Level Meeting in September 2011. This was also true from the perspective of the WHO Country Officer regarding the impact of the RAPIA and its results from a national and regional level with the government issuing a statement on access to medicines for NCDs. The Officer from the WHO Headquarters added “*as the IIF was able to substantively document the issue of the lack of access to medicines for NCDs, it was able to influence WHO and other stakeholders on this issue, including discussions within WHO on the issue of access to insulin and NCD medicines; the UN High Level Meeting on NCDs; Global Action Plan on NCDs and the inclusion of the 80% target on availability*”.

One aspect highlighted in the online questionnaire and interviews was the credibility of the research. This element is viewed as important by Stone et al. [33] and they link this to ‘peer review’, and that the individuals are linked to recognized institutions and are viewed as experts. For the RAPIA, this credibility was based on a mix of the people involved with the IIF, the past results of other RAPIA assessments, linking with trusted local partners, and the use of the RAPIA results by knowledge brokers such as the WHO and NCD Alliance. This not only gave credibility to the results as these were used by leading organizations, but also furthered the dissemination of results. Davis and Howden-Chapman [39] discuss the issue of transmitting the results of research versus these results being received and then actively used. This highlights the issue of ownership of data and how, in one country , the WHO and other local partners took ownership of the results using it in policy briefs and a presentation to the national assembly in 2010.

This was possible because of the methods used as well as the fact that this research was viewed as the first of its kind in terms of the issue studied and the number of countries where this research had taken place. One aspect that should not be neglected is the timing of this work from 2003 to 2010, a period during which NCDs and diabetes were gaining prominence on the global health agenda. Also of importance is the fact that the research was “grounded” in data that was relevant to the given country [19] and that this highlighted local problems enabling the development of local solutions (grounded in the local reality) [40]. This was facilitated by close collaboration between local partners and the IIF. Woelk et al. [35] mention the issues of trust, managing the political environment, and using champions at different levels to influence change. This was also discussed in having external versus internal people raise certain issues, but that the overall process was seen as a collaboration.

Despite the limitations of this work, the experience of the IIF using the RAPIA offers a unique perspective in which a similar research approach has been taken in six countries, allowing for insight into how health systems research can impact policy (Table 6). As with many studies assessing policy, this research only provides a snapshot due its methodological approach. The implementation of these recommendations would have been a good way to assess the impact of the RAPIA on policy. This was performed in Mozambique with a reassessment of the RAPIA showing some progress in terms of initial recommendations regarding access to insulin, healthcare worker training, access to diagnostics, and the increased role of the diabetes association, as well as the first national NCD strategy in sub-Saharan Africa [41]. Translation of research into policy is a complex process to understand; therefore, lessons from this study will be integrated into the WHO Manual How to Investigate Access to Chronic NCDs Care in Low- and Middle-income Countries [42], which is based on the RAPIA, to include the link between the research findings and policymakers from the beginning of such assessments. This approach has been piloted in Peru [43] to allow for a more transversal assessment to understand the link between this type of assessment and influence on policymakers at each step of the process. For the Global Action Plan, the WHO Officer at Headquarters felt that this new manual developed based on the initial RAPIA by the WHO and IIF “*will be an essential tool in the Monitoring and Evaluation of this target*” and by understanding links with policy, hopefully this will not only enable data to be collected, but for its use to be effective to inform and influence policymakers.

**Table 6:** **Key lessons from the implementation of the Rapid Assessment Protocol for Insulin Access in six low- and middle-income countries**

Lessons learnt	
–	Need for a formalized approach and robust methodology
–	Credibility of research team
–	Strong local partners
–	Research fills a clear gap in knowledge
–	Having context-specific results able to yield explicit recommendations
–	Need for a strong and comprehensive dissemination strategy involving a variety of local partners and strong local champions
–	Availability of results in peer reviewed publications, websites, and presentations
–	Importance of knowledge brokers, such as the WHO, and the need to influence these brokers in order to help inform policies

## Abbreviations

IIF: International Insulin Foundation
NCD: Non-communicable disease
RAPIA: Rapid Assessment Protocol for Insulin Access
WHO: World Health Organization
